# Activity in early visual areas predicts interindividual differences in binocular rivalry dynamics

**DOI:** 10.1152/jn.00509.2013

**Published:** 2013-12-18

**Authors:** Hiroyuki Yamashiro, Hiroki Yamamoto, Hiroaki Mano, Masahiro Umeda, Toshihiro Higuchi, Jun Saiki

**Affiliations:** ^1^Graduate School of Human and Environmental Studies, Kyoto University, Kyoto, Japan;; ^2^Department of Neurosurgery, Meiji University of Integrative Medicine, Kyoto, Japan; and; ^3^Department of Medical Informatics, Meiji University of Integrative Medicine, Kyoto, Japan

**Keywords:** binocular rivalry dynamics, continuous flash suppression, individual differences, early visual areas, functional magnetic resonance imaging, fMRI

## Abstract

When dissimilar images are presented to the two eyes, binocular rivalry (BR) occurs, and perception alternates spontaneously between the images. Although neural correlates of the oscillating perception during BR have been found in multiple sites along the visual pathway, the source of BR dynamics is unclear. Psychophysical and modeling studies suggest that both low- and high-level cortical processes underlie BR dynamics. Previous neuroimaging studies have demonstrated the involvement of high-level regions by showing that frontal and parietal cortices responded time locked to spontaneous perceptual alternation in BR. However, a potential contribution of early visual areas to BR dynamics has been overlooked, because these areas also responded to the physical stimulus alternation mimicking BR. In the present study, instead of focusing on activity during perceptual switches, we highlighted brain activity during suppression periods to investigate a potential link between activity in human early visual areas and BR dynamics. We used a strong interocular suppression paradigm called continuous flash suppression to suppress and fluctuate the visibility of a probe stimulus and measured retinotopic responses to the onset of the invisible probe using functional MRI. There were ∼130-fold differences in the median suppression durations across 12 subjects. The individual differences in suppression durations could be predicted by the amplitudes of the retinotopic activity in extrastriate visual areas (V3 and V4v) evoked by the invisible probe. Weaker responses were associated with longer suppression durations. These results demonstrate that retinotopic representations in early visual areas play a role in the dynamics of perceptual alternations during BR.

binocular rivalry (br) has been extensively investigated by neuroscientists exploring the neural substrates of visual awareness. Many electrophysiological and neuroimaging studies have revealed that activity at multiple levels of visual processing, from the thalamus to the visual cortex and beyond, correlate with perception during BR (Logothetis and Schall 1989; Leopold and Logothetis 1996; Tong et al. 1998; Polonsky et al. 2000; Tong and Engel 2001; Haynes and Rees 2005; Haynes et al. 2005; Wunderlich et al. 2005; Lee et al. 2005, 2007; Wilke et al. 2006; Maier et al. 2008; but see Watanabe et al. 2011). This suggests that the contents of visual awareness are represented at multiple neural sites. However, the crucial mechanism producing the perceptual switch itself remains unclear.

Psychophysical and modeling studies have suggested that both low- and high-level cortical processes underlie BR dynamics. Postulated low-level processes include sensory adaptation, suppression, and noise, whereas high-level processes include attention, perceptual decision, and inference (for review, see Blake and Wilson 2011). The multistage model of BR dynamics generation is consistent with recent results of transcranial magnetic stimulation (TMS) studies, which showed that single-pulse TMS over early visual areas (Pearson et al. 2007) and repetitive TMS over parietal regions (Carmel et al. 2010; Zaretskaya et al. 2010) modulated perceptual phase durations during BR.

The involvement of frontal and parietal regions for BR dynamics has also been repeatedly shown in functional neuroimaging studies (Lumer et al. 1998; Zaretskaya et al. 2010; Britz et al. 2011; but see Knapen et al. 2011), yet comparable evidence for early visual areas is not available. This might be due to the experimental paradigms employed in the previous studies; these studies generally localized brain activity relevant to spontaneous perceptual switches by contrasting brain activity that were accompanied by spontaneous perceptual alternations during real BR with those evoked by physical stimulus alternations that mimic BR. Such comparisons may not be appropriate for early visual areas, because these areas would respond to the physical alternations as strongly as or more strongly to the perceptual alternations (Lumer et al. 1998; Polonsky et al. 2000). Accordingly, time-locked activity to perceptual changes in early visual areas have been deemed nonspecific to BR in previous studies (e.g., Lumer et al. 1998).

The present functional magnetic resonance imaging (fMRI) study took a different approach to address the issue of the involvement of early visual areas for BR dynamics. Instead of focusing on brain activity around the time of perceptual switches, the present study highlighted brain activity during visual suppression, specifically, a retinotopic activity in early visual areas evoked by the onset of a suppressed stimulus. Our approach was motivated by two recent findings. The first is a close link between BR dynamics and sensory suppression; human psychophysics showed that longer awareness suppression is associated with stronger sensory suppression, measured as loss in visual sensitivity under continuous flash suppression (CFS; Tsuchiya et al. 2006). Under CFS, a less dynamic stimulus presented to one eye would be rendered invisible by the presence of a highly dynamic flashing stimulus presented to the other eye for a longer (10-fold) duration than conventional BR. At the same time, detection sensitivity for a contrast increment in the invisible stimulus decreased around sevenfold compared with conventional BR. We assume that the magnitude of sensory suppression can be measured by fMRI, since contrast increment thresholds can be predicted from retinotopic fMRI responses (Boynton et al. 1999). The second intriguing finding is the individual differences manifested in BR alternation rate (Pettigrew and Miller 1998) and its genetic influence (Miller et al. 2010), suggesting constitutional and stable differences in the processes underlying BR dynamics between individual brains.

Therefore, based on the findings of sensory suppression and individual differences in BR, we predict that, if a visual area played a role in BR dynamics, then its activity evoked by an invisible stimulus should be weaker in individuals with longer suppression durations and stronger in those with shorter suppression durations. This hypothesis was tested in the present study by rendering a probe stimulus invisible for as long as possible by CFS and then measuring retinotopic responses to the invisible probe using fMRI. We used CFS as a tool to probe BR dynamics, assuming that common neural processes underlie perceptual alternations during CFS and BR (see discussion for details).

## MATERIALS AND METHODS

### Subjects

Twelve subjects, including three of the authors, participated in this study (1 female, age range: 20–44 yr, median 26). All were experienced psychophysical observers. All, including the authors, were naïve to the purpose of the study, because the initial aim of this experiment was to make retinotopic maps using an invisible rotating wedge. All participated in the CFS experiment (range: 9–24 runs) and non-CFS control experiment (range: 4–8 runs), and six participated (*subjects S4*, *S6*, *S7*, *S9*, *S11*, and *S12*; see Fig. 3*B*) in the behavioral replay experiment (range: 5–22 runs). For each subject, experiments were conducted in two to three scanning sessions on different days. Before these experiments, all subjects participated in retinotopy experiments to define their visual areas and estimate their population receptive fields. All subjects had normal or corrected-to-normal visual acuity. Seven subjects had left-eye dominance and five had right-eye dominance as determined by Porta sighting tests (see Mendola and Conner 2007), in which a subject with both eyes open extends his/her arm and aligns the thumb with a distant target (i.e., the corner of a room) and then closes left and right eye alternately to determine the dominant eye in whose view the gap between the target and the thumb is smaller. All subjects provided written informed consent before participation. The local ethics committees at Kyoto University and Meiji University of Integrative Medicine approved the study.

### Visual Display

**Fig. 1.**
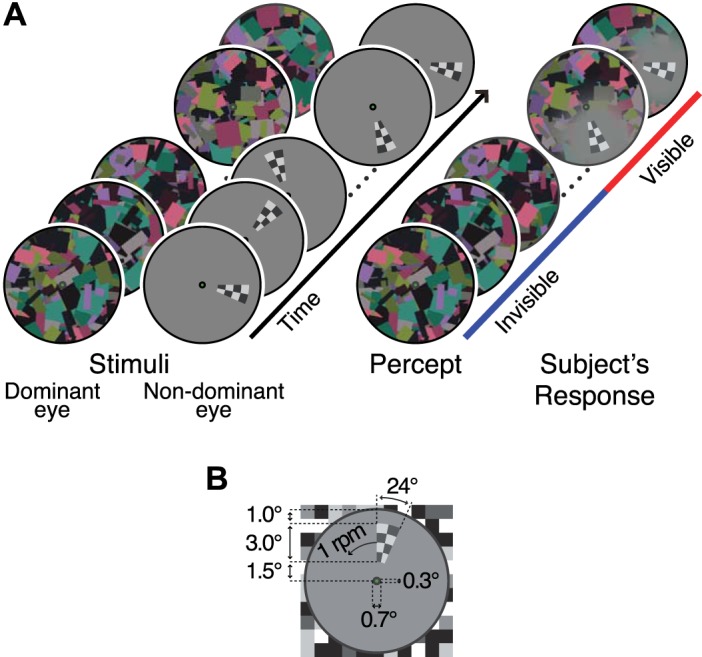
The visual stimuli used in the continuous flash suppression (CFS) experiment. *A*: high-contrast flashing Mondrian masks were presented to the dominant eye. The probe was a rotating checkerboard wedge presented to the nondominant eye. The visual awareness of the wedge fluctuated due to CFS. Subjects reported the visibility of the wedge continuously. *B*: size of the stimulus.

Visual stimuli were generated with an OpenGL-based in-house software running on a laptop computer (Evo N800w, Compaq) and projected onto a translucent screen by a Digital Light Processing (DLP) projector (U2-X2000, Plus, Japan, resolution 1,024 × 768, 60-Hz refresh rate), which was gamma-corrected using Mcalibrator2 software (Ban and Yamamoto 2013). The stimuli were presented dichoptically on the left and right halves of the screen placed above the subject's chin. Subjects viewed the dichoptic stimuli using custom-made prism glasses through an angled mirror positioned above the eyes, at a viewing distance of 21 cm. To divide both eyes' views, a septum was placed between the screen, the mirror, and the face. Each eye's view consisted of a gray annulus (36 cd/m^2^) with a fixation point at the center, and a black and white checkerboard surrounding the annulus (Fig. 1). The visual stimulus for each eye was presented within the annulus. The surrounding checkerboard aided stable binocular alignment.

### Main Experiments

#### CFS experiment.

In the CFS experiment, flashing Mondrians were presented to the subject's dominant eye and a probe stimulus was presented to the nondominant eye (Fig. 1), so that the probe would be likely to be suppressed due to CFS. The probe was a black and white checkerboard wedge (60% luminance contrast). It rotated smoothly counterclockwise around the fixation point at 1 rpm (6°/s). The use of the moving stimulus allowed us to probe onset responses without breaking CFS. To induce CFS, different Mondrian patterns were continuously flashed at 7.5 Hz. Each Mondrian pattern consisted of rectangles of random size (0.25–2.5°), orientation, and color (92% luminance contrast). Both stimuli were presented constantly throughout a 6 min 10-s run.

#### Subjects' task.

Subjects were instructed to report continuously on whether the wedge was completely invisible or visible, even if only a part of it was visible, by pressing one of two keys. Subjects were also instructed to maintain their fixation throughout the runs.

#### Non-CFS control experiment.

To measure individual intrinsic sensitivity to the visible wedge, we conducted the non-CFS control experiment, in which the Mondrians were replaced with a uniform background such that the wedge was always visible.

#### Replay experiment.

**Fig. 2.**
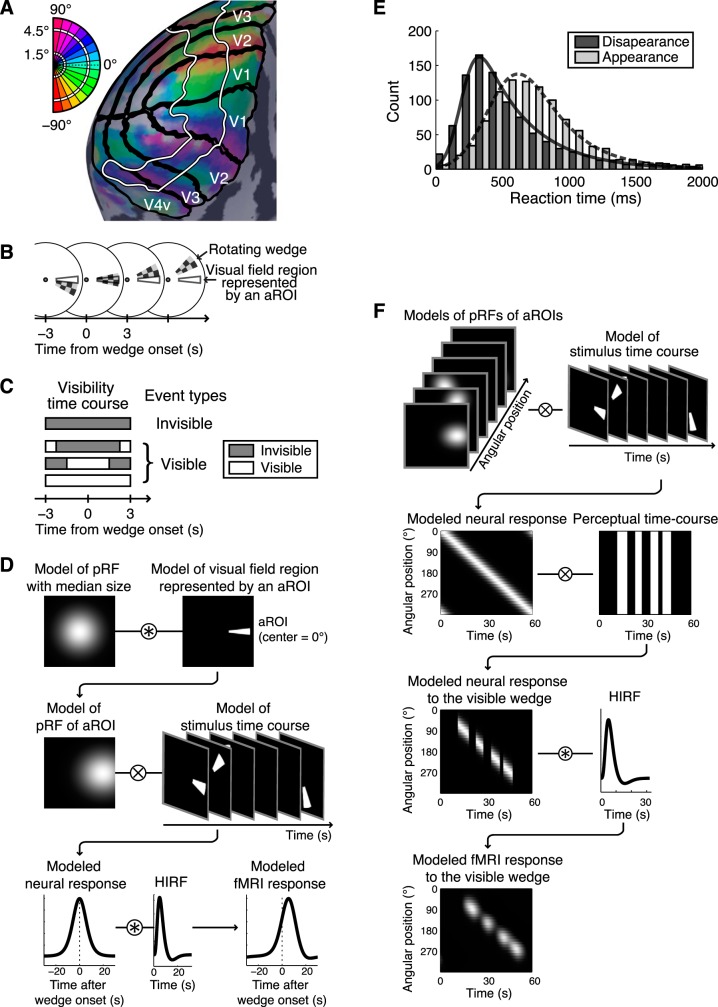
*A*: regions of interest (ROIs) shown on an inflated surface of one subject's left hemisphere. The black lines indicate the borders of visual areas. The cortical region surrounded by a white line retinotopically represents the visual field regions where the wedge travels. Colors on the surface represent the angular positions of estimated population receptive field (pRF) centers. Each color band represents angular region of interest (aROI). The colors in *top left inset* represent the corresponding angular positions in the visual field. *B*: schematic of the definition of wedge onset. The checkerboard wedge indicates the rotating wedge stimulus, and the region surrounded by a gray line indicates the visual field region represented by an aROI whose center is 0° (right horizontal meridian). 0 s Indicates the time of the wedge onset at a given aROI, which was defined as when the center of the wedge reached the center of the visual field region represented by the aROI. *C*: classification of the events. The gray and white horizontal bars indicate examples of subject's visibility time courses. The onsets were classified as invisible or visible, according to the subject's visibility reports while it traveled within the visual field region represented by the aROI (from 3 s before to 3 s after the onset). Unless the wedge was invisible during the travel, the event was classified as visible. *D*: model functional (f)MRI response incorporating the organization of pRF. For each aROI and visual area, a responsive visual field region of the aROI (pRF of aROI) was modeled by convolving the pRF model with median size with a model of visual field region represented by the aROI (*1st row*). A neural response in the aROI was modeled by summing the pixel-by-pixel product of the pRF of aROI and a stimulus model time course (*2nd row*). An fMRI response was modeled by convolving the individually estimated hemodynamic impulse response function (HIRF) to the modeled neural response (*3rd row*). The model fMRI response was fitted to the deconvolved response, and the height of the best fitting model was defined as the response amplitude. *E*: distribution of reaction times to the physical disappearance and appearance of the wedge measured in the replay experiment, pooled across 6 subjects. The solid and broken lines show the best-fitting ex-Gaussian curves for the disappearance and appearance, respectively. *F*: schematic illustration of contamination simulation. To model neural responses to the wedge, the individually modeled pRFs of aROIs were multiplied by a stimulus time course pixel-by-pixel and summed (*1st row*). Neural responses to the visible wedge were extracted according to the fluctuated perceptual time course for each run (*2nd row*). These neural responses were convolved with the individually estimated HIRF to synthesize fMRI responses to the visible wedge (*3rd row*). The simulated retinotopic responses to the visible wedge with spatial spreads and temporal fluctuations (*4th row*) were regressed out.

To estimate reaction time to the perceptual change, a replay experiment was conducted, in which the stimuli were physically modulated to simulate the perceptual time course recorded in the CFS experiment. To mimic the invisible phase, the wedge was physically removed. To mimic the visible phase, the wedge was presented to the nondominant eye and the contrast of the corresponding region of the Mondrian was decreased by a two-dimensional Gaussian window of full width at half maximum 5.5°, making the wedge visible. To mimic the perceptual switch, the contrasts of the stimuli were increased or decreased with a linear ramp of 200 ms. Subjects were instructed to report the visibility of the wedge as described in *Subjects' task*. Reaction time, from the end of the contrast increment or decrement to the key press, was measured. The reaction time data were pooled across subjects; reaction times longer than 2,000 ms were excluded. Median reaction times for the physical appearance and disappearance of the wedges were calculated separately (692 ms and 456 ms, respectively) and their distributions were modeled with ex-Gaussian functions (Fig. 2*E*; appearance: μ = 446.5, σ = 186.6, τ = 297.4; disappearance: μ = 199.8, σ = 94.6, τ = 356.0; for details on ex-Gaussian function see Lacouture and Cousineau 2008; Saiki et al. 2011). The median reaction times were subtracted from the raw key press time courses to compensate for the delay between the perceptual change and the key press in the following fMRI analysis. The distributions of the reaction times were used in a simulation described below (see *Contamination control*).

### Imaging Data Acquisition

Functional images were acquired using a 1.5T scanner (Signa, General Electric, Milwaukee, WI) with a surface coil placed at the occipital pole by T2*-weighted BOLD-sensitive gradient-echo echo-planar imaging [repetition time (TR) = 2000 ms; echo time (TE) = 50 ms; flip angle = 90°; field of view (FOV) = 200 × 200 mm; matrix 128 × 128; slice thickness 4 mm; voxel size 1.56 × 1.56 × 4 mm; 16 slices perpendicular to the calcarine sulcus, covering the occipital cortex]. For each run, 185 volumes were acquired. For each scanning session, T1-weighted structural images were acquired for anatomical registration.

### Defining the Visual Areas and Regions of Interest

#### Estimation of hemodynamic impulse response function.

Hemodynamic impulse response function (HIRF) was modeled by a canonical hemodynamic response function of SPM5 (http://www.fil.ion.ucl.ac.uk/spm) with parameters estimated separately for each subject. The parameters were estimated from the fMRI responses measured in HIRF runs. The HIRF runs were conducted in the same scanning sessions as for the CFS experiment. Subjects participated in at least four runs. In the HIRF runs, a black and white checkerboard ring (eccentricity 1.5–4.5°, luminance contrast 92%, flickered alternately at 4 Hz) was presented for 15 s and then disappeared for 15 s. This stimulus cycle was repeated for 12 times during a run. fMRI time courses from voxels, whose response time courses were strongly correlated with the stimulus alternations (*r* > 0.6, within 0–12 s time lag), were averaged. The averaged fMRI time course was then subjected to a nonlinear least squares fitting procedure to search the best fitting HIRF parameters.

#### Retinotopic mapping and identification of visual areas.

Retinotopic visual areas V1, V2, V3, and V4v were identified using standard phase-encoding retinotopy measurements for each subject (Sereno et al. 1995; DeYoe et al. 1996; Engel et al. 1997). The details of the measurements and surface mapping have been described elsewhere (Yamamoto et al. 2008, 2012).

#### Estimation of population receptive field.

A population receptive field (pRF) is a region of visual space that activates a population of neurons residing at a point on the cortical surface when it is stimulated. The pRF was estimated using a two-dimensional Gaussian model, which is similar to that described by Dumoulin and Wandell (2008). The pRF model was defined by position and size parameters, specifically, the coordinates of its center in the visual field and the standard deviation (σ) of the Gaussian. These parameters were estimated using the fMRI responses to the rotating wedge and expanding ring during the retinotopy experiments. Briefly, a model neural response of a given pRF model at a given time point was computed by multiplying the pRF model, which is represented as an image, and a stimulus model, which is a series of images representing the stimulated regions in the visual field in a pixel-by-pixel manner and then summing the products. The neural response was then convolved with the HIRF estimated for each subject, yielding a model fMRI response. This model response was fitted to the measured fMRI response by a grid-search algorithm, giving the best fitting pRF parameters. The cortical point, the response of which yielded a correlation coefficient with the best fitting model response <0.5, was excluded from the following analysis.

### Behavioral Data Analysis

For each subject, the median and mean phase durations for wedge dominance (visible) and suppression (invisible) were calculated from the key press time course recorded in the CFS experiment. To characterize the statistical distributions of the phase durations, we fitted gamma and lognormal distributions that BR phase durations typically follow (Levelt 1967; Lehky 1995; Zhou et al. 2004). The goodness of the fits was assessed with the Kolmogorov-Smirnov test (K-S test). To reject artifacts, key presses with durations <250 ms were removed from the analysis. To compute confidence intervals of the median across perceptual phases for each subject, the bootstrapping technique was used (Efron and Tibshirani 1993). In the bootstrap, phase durations were randomly sampled with replacement from the all the visible or invisible phase durations of each observer. Then, the medians of the resampled durations were calculated. This procedure was repeated 10,000 times. Thereby, the 95% confidence intervals were computed from the resulting distributions.

### fMRI Data Analysis

The fMRI data were analyzed using in-house software written in C and Matlab (Yamamoto et al. 2008, 2012).

#### fMRI signal sampling and preprocessing.

After correcting for motion and slice timing, the functional images were registered to the cortical surface of each subject. The fMRI data were sampled independently from each visual area. Voxels shared by multiple visual areas were excluded from the sampling, so that the sampled signals would not be contaminated by signals from other areas. fMRI data from the sampled voxels were subjected to voxel-based preprocessing, which included discarding the initial (10 s) signal to minimize magnetic saturation effects, removing linear trend, and converting to percent signal change.

#### Angular regions of interest.

For each visual area, the fMRI time course was analyzed according to the polar angle representation of the visual field (Fig. 2*A*), which is referred to as an angular region of interest (aROI). Specifically, according to angular positions of the pRF centers, the surface of each visual area was divided into 30 subregions, each of which represents a 12° polar angle in the visual field (Fig. 2*A*, *top left inset*). The subregions were further restricted to the retinotopic representation of the visual field region where the wedge travels (1.5–4.5°; Fig. 2*A*, the regions surrounded by a white line), based on the eccentric positions of the pRF centers.

#### Retinotopic responses to the invisible wedge.

We focused on retinotopic responses to the invisible wedge (i.e., the transient responses evoked by the onset of the rotating wedge when it entered a given pRF; see Fig. 2*B*), in contrast to those measured in conventional fMRI studies of BR in which stimuli were constantly present in pRFs (e.g., Polonsky et al. 2000; Wunderlich et al. 2005). Our fMRI measurements can therefore be interpreted as the neuronal counterpart of psychophysical measurements of suppression depth using probe thresholds (e.g., Tsuchiya et al. 2006; Alais et al. 2010).

The mean retinotopic response time courses to the invisible wedge were estimated using a deconvolution technique (Dale 1999), which is essentially equivalent to a selective averaging with corrections for temporally overlapping responses. In the deconvolution analysis, the design matrix was defined to isolate the response to the invisible wedge from the signals caused by other confounds, the retinotopic responses to the visible wedge, response modulations around the time of perceptual appearance and disappearance of the wedge, which we call transition-related responses, and the constant terms. The onset time of the wedge for a given aROI was defined as when the center of the wedge reaches the center of the aROI (Fig. 2*B*). These onsets were classified as invisible or visible, according to the subject's visibility reports while it traveled within the visual field region represented by the aROI (from 3 s before to 3 s after the onset; Fig. 2*B*). To maximize the isolation, the onset was classified as invisible only if the wedge was invisible throughout the travel; it was classified as visible otherwise (Fig. 2*C*). Note that, with this criterion, the invisible phases <6 s would be classified as visible events, because the wedge takes 6 s to go through the visual field region represented by an aROI. The deconvolution time window was set from 20 s before to 40 s after the onset.

The transition-related responses were added to regress out the responses evoked around the time of perceptual transitions that are irrelevant to the onset of the visible and invisible wedge, such as responses that would covary with the contrast of the perceived image (Polonsky et al. 2000; Wunderlich et al. 2005) or the transient responses at the time of perceptual switches (Lumer et al. 1998; Polonsky et al. 2000; but see Haynes and Rees 2005 for discussion whether the response is transient or sustained). To minimize their effects on the retinotopic responses to the wedge, the regressors for the transition responses were defined independently of the position of the wedge (i.e., nonretinotopically, so that all the aROIs would respond equally to a perceptual switch). They were modeled separately for perceptual appearance (invisible to visible) and disappearance (visible to invisible). The time window was set from the beginning of the perceptual phase just before the transition to the end of the phase just after the transition (limited at most from 30 s before to 30 s after the transition). The design matrices were generated for each aROI and run. To increase the signal-to-noise ratio, theses matrices were concatenated into one matrix, on the assumption that responses are uniform across aROIs and runs. Then, for each subject, the deconvolution was carried out for fMRI signals concatenated across aROIs and runs. Note that pRF sizes in the aROIs were not taken into account at this stage.

#### Response amplitudes.

Next, we estimated the amplitudes of the retinotopic responses by fitting a model waveform incorporating the organization of pRF (Fig. 2*D*). First, the median pRF size across aROIs was computed for each visual area and subject (average of the medians across subjects: V1: σ = 0.8°; V2: σ = 1.1°; V3: σ = 1.8°; V4v: σ = 2.7°). Second, the wedge-shaped visual field region represented by an aROI was convolved with the median pRF model (Fig. 2*D*, *1st row*), generating a model of a responsive visual field region of an aROI; that is, a pRF of aROI. Third, the pRF of aROI was multiplied by a stimulus model time course pixel-by-pixel and summed to generate a model neural response (Fig. 2*D*, *2nd row*) in the similar way as described in *Estimation of population receptive field*. The neural response was convolved with the individually estimated HIRF to generate a model fMRI response (Fig. 2*D*, *3rd row*). Finally, the response amplitudes were estimated as the height of the model response that fit best to the measured responses. In addition to the fitted amplitude, we used a peak amplitude, which was calculated by subtracting baseline intensity from peak intensity. The peak intensity was defined by the average intensity of three time points around the maximum within 12 s after the onset. The baseline intensity was defined by the average intensity of three time points around the minimum from 12 s before to 2 s before the onset.

The bootstrapping technique (Efron and Tibshirani 1993) was used to obtain confidence intervals of the response time courses and amplitudes for each visual area and subject. A single bootstrap sample was made as follows. First, we randomly chose runs with replacement from all the runs of each subject. Then, from the fMRI and behavioral data sets of the chosen runs, we deconvolved fMRI responses time courses and estimated the response amplitudes in the same way as for the original data sets. This procedure was repeated 10,000 times, and then 95% confidence intervals were obtained from the resulting distribution.

#### Correlation analysis.

To characterize the relationship between BR dynamics and the activity in early visual areas, we computed Spearman's rank correlation coefficient (ρ) between the median suppression durations and the response amplitudes to the invisible wedge for each area. The statistical significance of the correlation was calculated via a two-tailed permutation test.

### Control Analyses

#### Contamination control.

The retinotopic responses to the wedge would spread spatially over the cortical surface due to the pRFs. In addition, the responses were temporally fluctuated, because of the fluctuation of the reaction time between the perceptual change and the key press. These response spreads might contaminate spatially and temporally close responses (i.e., responses to temporally close onsets in neighboring aROIs). However, such spreads were not modeled in the deconvolution analysis; therefore, the estimated responses to the invisible wedge should be contaminated to some extent by those to the visible wedge. To correct for the possible contamination, we simulated the retinotopic responses to the visible wedge with spatial spreads and temporal fluctuations and regressed them out in the contamination control analysis as follows (Fig. 2*F*).

First, for each subject and run, a perceptual time course for the visibility of the wedge was generated from the key press recorded in the CFS experiment. To simulate the reaction time fluctuation, the timing for each perceptual switch was shifted back by a random duration sampled from the reaction time distributions estimated in the replay experiment (Fig. 2*E*). Second, to model neural responses to the wedge, the individually modeled pRFs of aROIs were multiplied by a stimulus time course pixel-by-pixel and summed (Fig. 2*F*, *1st row*), as described in *Response amplitudes*. Third, from the modeled neural responses, neural responses to the visible wedge were extracted according to the fluctuated perceptual time course for each run (Fig. 2*F*, *2nd row*). Fourth, these neural responses were convolved with the individually estimated HIRF to synthesize fMRI responses to the visible wedge (Fig. 2*F*, *3rd row*). The simulated retinotopic responses to the visible wedge with spatial spreads and temporal fluctuations (Fig. 2*F*, *4th row*) were then included in the design matrix of the deconvolution analysis described above, replacing the regressor for the retinotopic response to the visible wedge. With the use of this design matrix, the responses to the invisible wedge were deconvolved, regressing out the contamination. Finally, the resulting response amplitudes were used to recalculate the correlation coefficients with the median suppression durations.

#### Signal-to-noise ratio control.

The total number of the invisible onsets, from which the responses to the invisible wedge were derived, differed substantially across subjects (V1: range: 225–2,672, median: 1,164; V2: range: 230–2,381, median: 848; V3: range: 292–2,509, median: 1,060; V4v: range: 135–1,986, median: 690.5). The number of the onsets (i.e., samples) would directly affect the signal-to-noise ratio (SNR) of the estimated responses. As the total number of the invisible onsets tended to be smaller in subjects with short suppression durations compared with subjects with long ones, this imbalance between subjects might affect the correlation between the fMRI responses and the suppression durations. If there were a systematic bias in the response amplitudes depending on the number of the onsets, such that the smaller number of samples would lead to the larger amplitudes, the correlation could be explained by the imbalance of the samples. To control for the imbalance, we performed a SNR control analysis on the data collected in the CFS experiments, in which the total number of the invisible onsets was equated across subjects. Specifically, we subsampled the invisible onsets randomly without replacement, so that the total number for each subject matched that of the fewest subject. Then, the responses to the subsampled onsets were deconvolved and the amplitudes were estimated in the same way as for the original data, while the responses to the rest of the invisible onsets were regressed out. This procedure was iterated 100 times, and the estimated amplitudes were averaged across iterations. Finally, the averaged amplitudes were used to recalculate the correlation coefficients.

#### Partial correlation analysis to control for the effect of the duration of the suppression periods.

The correlation between the individuals' median suppression durations and the fMRI responses might not be due to the stable characteristics of the individuals; instead, it might be due solely to the duration of the suppression periods itself, regardless of the individual differences. If this is true then, for example, when the fMRI responses were derived from equally long suppression periods, the fMRI responses of subjects with long suppression periods would be as strong as those of subjects with short suppression periods. To explore the neural correlates of the individual differences in perceptual dynamics during BR, controlling for the effect of the duration of the suppression itself, we performed a partial correlation analysis. Specifically, for each subject, the suppression periods within the range from 6 to 34 s were divided into seven bins of 4-s wide. Within this range, data from most of the subjects were available (mostly from 11 subjects; data from at least 7 subjects). For each bin, fMRI responses to the invisible wedge were deconvolved and their amplitudes were estimated in the same way as for the original data. Then, data from all the bins were combined and partial Spearman's correlation coefficients (ρ) between the fMRI responses and individuals' median suppression durations were computed, regressing out the influence of the duration of the suppression periods.

## RESULTS

### Suppression Durations Varied Widely Across Subjects

**Fig. 3.**
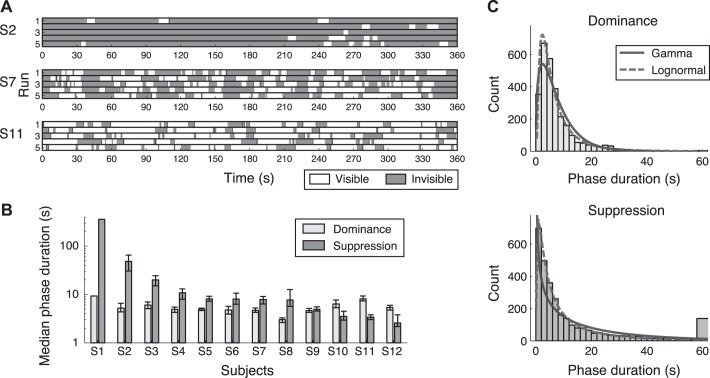
Behavioral data. *A*: examples of perceptual time courses from 3 representative subjects. The first is a subject with long suppression (*top row*, *S2*), the second is a subject with intermediate suppression (*middle row*, *S7*), and the third is a subject with short suppression (*bottom row*, *S11*). The difference in the duration of the suppression (invisible) periods and the frequency of the perceptual switching is clearly shown. *B*: individuals' median dominance and suppression phase durations. The error bars are bootstrap 95% confidence intervals of the median. There were ∼130-fold differences between subjects in suppression durations and ∼3-fold differences in dominance durations. *C*: histograms of dominance and suppression phase durations pooled across 12 subjects. The distribution of the suppression durations had a much longer tail than that of the dominance durations. These distributions were well approximated by a lognormal distribution (broken line) but not by a gamma distribution (solid line).

Subjects' perceptions fluctuated while viewing the dichoptic stimuli consisting of the rotating wedge and the flashing Mondrians. Figure 3*A* shows examples of perceptual time courses from three representative subjects (*S2*, *S7*, and *S11*). Based on the visibility reports for the wedge, medians of suppression and dominance durations were calculated for each subject (Fig. 3*B*). On average, suppression phases (average of medians: 40.4 s) were about seven times longer than dominance phases (average of medians: 5.6 s), showing the characteristic of CFS (Tsuchiya and Koch 2005). Notably, there were ∼130-fold differences between subjects in suppression durations (medians range: 2.6 to 360 s; Fig. 3*B*). There were approximately threefold differences between subjects in dominance durations (medians range: 3.0 to 9.3 s).

Interestingly, five of the subjects (*S3*, *S5*, *S7*, *S10*, and *S12*) show a periodic pattern that synchronized with the rotation of the wedge (60 s) (Fig. 3*A*, *middle row*). This periodic pattern of visibility might be due to an onset rivalry bias, which is dependent on the visual field position (Carter and Cavanagh 2007; Knapen et al. 2009; Stanley et al. 2011), so that when the wedge reached a particular position of the visual field (i.e., at the onset at that position), the wedge tended to be seen. However, we do not discuss this further here, because this periodicity was observed in both types of subjects with long and short suppression durations, suggesting that the onset bias occurred independently of the suppression duration.

Figure 3*C* shows the histograms of dominance and suppression phase durations pooled across subjects. These distributions, both skewed positively, were well approximated by a lognormal distribution, suggesting that they followed typical BR phase duration distributions (Lehky 1995; Zhou et al. 2004). A K-S test confirmed that these distributions were not significantly different from lognormal distribution (suppression: μ = 1.80, σ = 1.38, K-S statistic = 0.02, *P* = 0.079; dominance: μ = 1.65, σ = 0.89, K-S statistic = 0.02, *P* = 0.069; uncorrected). Gamma distribution, which is probably the most popular distribution used to describe phase durations in BR (Levelt 1967), did not fit well (suppression: α = 0.59, β = 29.26, K-S statistic = 0.13, *P* < 0.001; dominance: α = 1.46, β = 5.19, K-S statistic = 0.08, *P* < 0.001; uncorrected). This better fit of lognormal distribution was also true for individual data, especially for suppression durations. This might be due to a strong suppressive effect of CFS, compared with conventional BR.

### Invisible Wedge-Evoked Retinotopic Responses

**Fig. 4.**
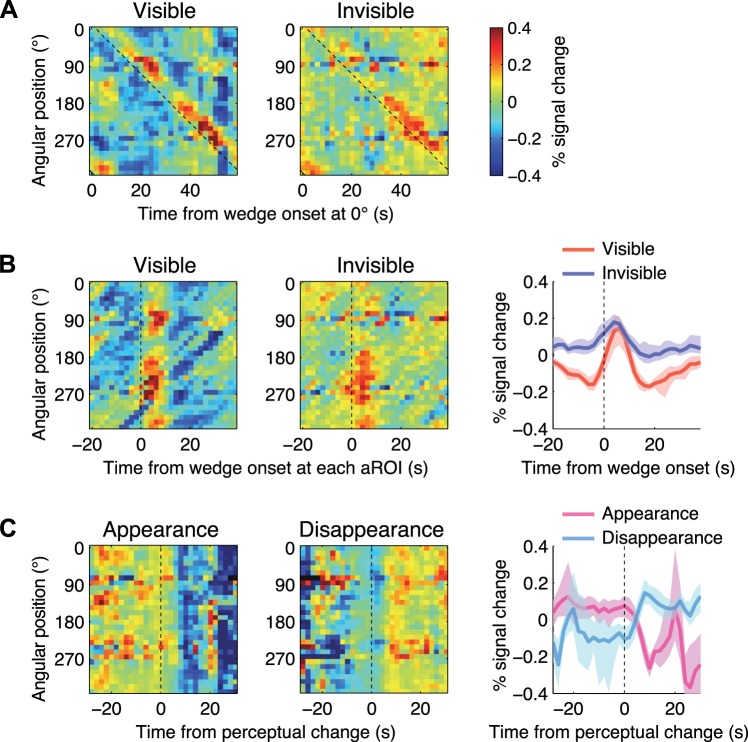
Selectively averaged event related responses. *A*: spatiotemporal plot of retinotopic fMRI responses in V1 averaged across subjects (*n* = 12) after averaging selectively during the visible (*left*) and invisible (*right*) phases for each subject, keeping the relative time during the stimulus cycle. The vertical axis represents angular position in the visual field, and the horizontal axis represents time after the onset of the wedge at the right horizontal meridian in the visual field (0°). Each horizontal trace shows the averaged fMRI time course within the corresponding aROI. The diagonal broken line indicates the time of the wedge onset. *B*: shifted version of the selectively averaged retinotopic responses in V1 averaged across subjects (*left* and *middle*). The response time courses were shifted to align the onsets across aROIs and subjects. The horizontal axis represents time after the wedge onset at each aROI. The broken line indicates the time of the wedge onset. *Right*: waveforms of the retinotopic responses averaged across aROIs. The shaded regions represent bootstrap 95% confidence intervals of the mean across subjects. *C*: spatiotemporal plot of transition-related fMRI responses in V1 averaged across subjects (*n* = 12) after averaging selectively around the time of the 2 perceptual transitions: appearance (invisible to visible; *left*) and disappearance (visible to invisible; *middle*). The horizontal axis represents time after the perceptual change. *Right*: waveforms of the transition-related responses averaged across aROIs and subjects. The shaded regions represent bootstrap 95% confidence intervals of the mean across subjects. Note that these patterns of transition responses are consistent with previous fMRI studies of conventional BR (see results).

We found two kinds of activity in visual areas V1, V2, V3, and V4v during CFS. The first was the periodic activity that synchronized with the rotation of the wedge. Figure 4*A* shows spatiotemporal fMRI responses in V1, which were averaged selectively during the visible (Fig. 4*A*, *left*) or invisible (Fig. 4*A*, *righ*t) periods for each subject and then averaged across subjects. In these plots, each horizontal trace shows the fMRI time course within a corresponding isoangular band (aROI). The diagonal pattern from the *top left* to the *bottom right corner* of the image indicates that each aROI responded when the wedge arrived at the visual field position represented by the aROI (Fig. 4*A*, diagonal broken line), demonstrating the retinotopic activity evoked by the visible and invisible wedge. These responses were shifted to make the onsets aligned across aROIs (Fig. 4*B*, *left* and *middle*) and then averaged across aROIs to obtain waveforms (Fig. 4*B*, *right*). These waveforms clearly show that V1 responded time locked to the onset of the invisible wedges (blue waveform in Fig. 4*B*, *right*) as well as to that of the visible one (red waveform in Fig. 4*B*, *right*). Similar patterns of retinotopic responses were observed in the other areas.

The second type of the activity was time locked to when the subjects reported that the invisible wedge became visible or the visible wedge became invisible. Figure 4*C* shows spatiotemporal fMRI responses around the time of the perceptual switches, averaged separately for appearance and disappearance, and averaged across subjects. When the wedge became visible (appearance), activity in V1 decreased (Fig. 4*C*, *left*, and magenta waveform at *right*), and when the wedge became invisible (disappearance) activity in V1 increased (Fig. 4*C*, *middle*, cyan waveform at *right*), demonstrating the presence of the transition-related responses. It should be noted that these transition-related responses are consistent with previous reports that showed fMRI responses in early visual areas and the lateral geniculate nucleus correlated with contrast of the perceived image during BR (Polonsky et al. 2000; Wunderlich et al. 2005). The response decreased when the perceived contrast decreased as the wedge (lower contrast) became visible and the corresponding part of the flashing mask (higher contrast) disappeared, and the response increased when the perceived contrast increased as the wedge disappeared and the whole part of the flashing mask became visible. Similar patterns of transition-related responses were observed in the other areas.

**Fig. 5.**
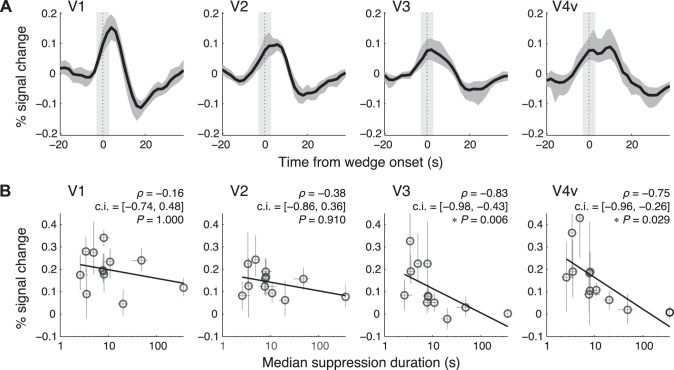
Deconvolved retinotopic responses and correlations between the responses and the suppression durations. *A*: retinotopic responses evoked by the onset of the invisible wedge averaged across subjects after deconvolution for each subject. The horizontal axis represents the time from the wedge onset at aROIs. The light gray regions indicate the duration of the stimulation for the aROIs. The dark gray regions represent bootstrap 95% confidence intervals of the mean across subjects. Note that the responses in all areas rose before the onset (time = 0), because of the definition of the onset (see Fig. 2*B*) and the pRF of aROI (see Fig. 2*D*). *B*: scatterplots of the amplitudes of the retinotopic responses to the invisible wedge and the median suppression durations measured in the CFS experiment. Each symbol represents a subject (*n* = 12). Horizontal error bars indicate bootstrap 95% confidence intervals (c.i.) of the median suppression durations, and vertical error bars indicate bootstrap 95% confidence intervals of the estimated amplitudes. Spearman's correlation coefficients (ρ), bootstrap 95% confidence intervals of ρ, and Bonferroni corrected *P* values are shown (*P < 0.05).

**Fig. 6.**
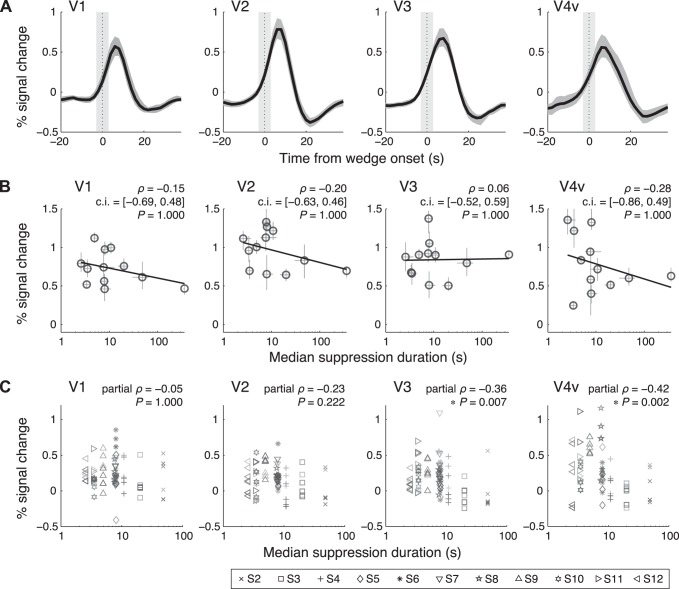
*A*: retinotopic responses to the visible wedge during the non-CFS experiment averaged across subjects after deconvolution for each subject. The conventions follow Fig. 5*A*. *B*: scatterplots of the amplitudes of the responses to the visible wedge measured in the non-CFS experiment and the median suppression durations measured in the CFS experiment. The conventions follow Fig. 5*B*. *C*: partial correlations between the response amplitudes to the invisible wedge derived from equally long suppression periods (7 bins with 4 s wide, from 6 to 34 s) and the individuals' median suppression durations measured in the CFS experiment. Each symbol type represents the data of 1 subject (*n* = 11). The brightness of the symbols codes the duration of the suppression periods from which the responses were derived: lighter colors represent responses from shorter suppression periods and vice versa. Partial correlation analysis confirmed the significant correlations between the fMRI responses and individuals' median suppression durations, after removing the potential contributions of the duration of the suppression periods itself. Partial Spearman's correlation coefficients (ρ), and Bonferroni corrected *P* values are shown.

Since these transition-related responses (Fig. 4*C*) temporally overlapped with the responses during suppression or dominance periods (Fig. 4*B*), they certainly contaminated the retinotopic responses to the invisible wedge (blue waveform in Fig. 4*B*, *right*), which was of interest in this study. We therefore isolated the retinotopic response to the invisible wedge by regressing out the other components in the deconvolution analysis. The data in Fig. 5*A* show the time course of the isolated retinotopic responses to the invisible wedge pooled across aROIs and averaged across subjects. We found robust retinotopic activity to the invisible wedge in all areas. The response amplitudes were significantly larger than zero [one tailed *t*-test, *P*-values were Bonferroni-corrected for the four visual areas tested; V1: *t*_(11)_ = 7.63, *P* < 0.001; V2: *t*_(11)_ = 8.51, *P* < 0.001; V3: *t*_(11)_ = 3.54, *P* = 0.009; V4v: *t*_(11)_ = 4.38, *P* = 0.002]. Note that the responses in all areas rose before the onset (time = 0 in Figs. 5*A* and 6*A*), because the onset of the wedge was defined as the time when the center of the wedge reached the center of the visual field region represented by the aROI (see Fig. 2*B*), and furthermore this visual field region was broadened by the pRFs (see Fig. 2*D*, *2nd row*).

### Individual Differences in Suppression Durations Were Negatively Correlated with Retinotopic Responses to the Invisible Wedge

Our goal was to determine if the degree of neural suppression in an individual's brain contributes to the variability in BR dynamics. To this end, we analyzed the relationship between the retinotopic responses to the invisible wedge and the median suppression durations. Figure 5*B* plots each subject's response amplitude vs. his/her suppression duration for visual areas V1, V2, V3, and V4v. We found that in V3 and V4v, weaker responses were associated with longer suppression durations. These correlations were statistically significant in V3 and V4v (Fig. 5*B*). We obtained qualitatively similar result by using the mean suppression durations instead of the median (V1: ρ = −0.08, *P* = 1.000; V2: ρ = −0.47, *P* = 0.510; V3: ρ = −0.81, *P* = 0.009; V4v: ρ = −0.94, *P* < 0.001; *n* = 12; Bonferroni corrected) and by using the peak amplitudes instead of the fitted amplitudes (V1: ρ = −0.18, *P* = 1.000; V2: ρ = −0.41, *P* = 0.770; V3: ρ = −0.71, *P* = 0.048; V4v: ρ = −0.73, *P* = 0.040; *n* = 12; Bonferroni corrected). Such a correlation was not found for the median dominance durations (V1: ρ = −0.36, *P* = 1.000; V2: ρ = −0.36, *P* = 0.985; V3: ρ = −0.17, *P* = 1.000; V4v: ρ =−0.15, *P* = 1.000; *n* = 12; Bonferroni corrected).

We performed a series of control analyses to confirm the results. The first is the sensitivity control, in which we assessed if the extracted responses to the invisible wedge merely reflected the individual's intrinsic sensitivity, rather than his/her sensitivity to the suppressed wedge. If this is the case, then retinotopic responses to the visible wedge measured in the non-CFS experiment (Fig. 6*A*), in which the Mondrian masks were removed, should also predict the suppression durations measured in the CFS experiment. However, the responses to the visible wedge failed to predict the suppression duration in all areas (Fig. 6*B*).

The second is the contamination control, in which we tested if the correlations were due to the responses to the visible wedge, rather than the invisible one. For subjects with shorter suppression durations, the perceptual switches were more frequent and, therefore, the responses to the invisible wedge would be more likely to be contaminated from those to the visible wedge. Given that a visible stimulus would evoke a larger response than an invisible one, as reported for the tool images (e.g., Hesselmann and Malach 2011), it is possible that the correlation can be accounted for by the contamination. To rule out this possibility, we simulated the responses to the visible wedge with spatial spread due to the pRFs and with temporal spread due to the reaction time fluctuations and then regressed out these contaminating components. After the contamination was removed, the correlations in V3 and V4v remained statistically significant (V1: ρ = −0.15, *P* = 1.000; V2: ρ = −0.38, *P* = 0.874; V3: ρ = −0.79, *P* = 0.014; V4v: *ρ* = −0.86, *P* = 0.002; *n* = 12; Bonferroni corrected).

The third is the SNR control, in which we ruled out the potential contribution from the imbalance of the SNR between subjects, which arose from the difference in the number of the onsets of the invisible wedge. After equalizing the SNR between subjects by subsampling the invisible onsets, we again found the significant correlation between the fMRI responses and the suppression durations in V3 and V4v (V1: ρ = −0.21, *P* = 1.000; V2: ρ = −0.38, *P* = 0.910; V3: ρ = −0.78, *P* = 0.019; V4v: ρ = −0.76, *P* = 0.024; *n* = 12; Bonferroni corrected).

Finally, we controlled for the potential effect of the duration of the suppression periods itself. The observed correlation might not be due to the individual differences in the perceptual dynamics during BR but might be due solely to the duration of the suppression periods itself from which the fMRI responses were extracted. To rule out this possibility, we performed a partial correlation analysis on the fMRI responses derived from seven bins of equally long suppression periods between subjects (collected from all subjects except subject S1). The partial correlation analysis confirmed that the fMRI responses in V3 and V4v were significantly correlated with the individuals, when the influence of the duration of the suppression period itself was removed (F ig. 6*C*).

## DISCUSSION

In the present study, we showed a close link between individual's perceptual dynamics during CFS and activity in early visual areas. The present experiment showed that early visual areas responded to the moving checker stimulus in a retinotopic manner, even when it was rendered invisible by CFS. Crucially, the magnitude of the retinotopic responses predicted the perceptual dynamics of individuals. Subjects with weaker extrastriate responses in V3 and V4v had longer suppression durations.

To our knowledge, the present study is the first to report a significant association between neural activity and interindividual differences in the dynamics of CFS and, in a broader sense, BR. The association was found in early visual areas, which is consistent with recent TMS and magnetic resonance spectroscopy studies. Pearson et al. (2007) showed that TMS over early visual areas shortens phase durations during BR. van Loon et al. (2013) showed that the GABA concentration in the visual cortex was correlated with interindividual differences of perceptual dynamics of bistable perception including BR. Our results bridged the gap between magnetic stimulation and behavior and the gap between neurotransmitter and behavior by showing tight coupling between brain activity and behavior. The neural activity retinotopically representing the suppressed stimulus were indeed strongly suppressed in retinotopic visual areas (V3 and V4v) of subjects with longer suppression. Taken together, these findings suggest that if, in early visual areas, there is abundant GABA and the neural representation of the suppressed stimulus receives a lot of inhibitory input so that its activity is greatly suppressed, the suppression lasts for a long time.

The activity in early visual areas has been suggested to represent the contents of visual awareness by previous neuroimaging studies demonstrating that the activity correlated with the alternating percept during BR (Polonsky et al. 2000; Tong and Engel 2001; Haynes and Rees 2005; Haynes et al. 2005; Wunderlich et al. 2005; Lee et al. 2005, 2007). Our results suggest that the role of early visual areas in BR is not solely to represent the contents of the percept. They also contribute to the perceptual dynamics of BR.

Previous imaging studies have suggested the involvement of higher level cortical areas in rivalry dynamics, including the parietal and frontal cortices. An fMRI study by Lumer et al. (1998) found that activation of the right fronto-parietal network was time locked to the spontaneous perceptual alternation in BR. A recent structural imaging study by Kanai et al. (2010) showed that anatomical features of the bilateral parietal cortex could account for the interindividual variability in alternation rate during bistable figure perception. The present findings, together with previous ones, confirm that both low-level visual sensory regions and higher level executive regions are involved in determining perceptual dynamics in BR.

**Fig. 7.**
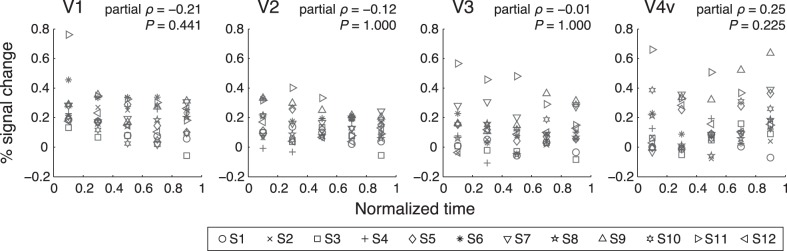
Response amplitudes to the invisible wedge as a function of timing of the invisible wedge onset. Horizontal axis represents normalized time in proportion to duration of given suppression period. Each symbol type represents the data of 1 subject (*n* = 12). Partial correlation analysis revealed no significant correlations between the response amplitudes and the relative onset timing, after removing the influence of the subject. Partial Spearman's correlation coefficients (ρ) and Bonferroni corrected *P* values are shown.

A recent psychophysical study demonstrated that the perceptual dynamics of BR was shaped by neural adaptation and reciprocal inhibition (Alais et al. 2010). They showed that visual sensitivity to brief probe stimuli changed over time during an episode of suppression. If such adaptation process also played a key role in our experiment, the responses to the invisible stimulus should have increased over time during a suppression period as they reported. To test this notion, we performed an additional analysis on the data collected in the CFS experiment. In brief, we classified invisible onsets included in each suppression period into five time bins according to their timing relative to the duration of that suppression period, and then we deconvolved retinotopic responses for each bin. Contrary to our prediction, the response amplitudes to the invisible wedge did not change over time during a suppression period; no significant correlation was observed between the amplitudes and the onset timing in all areas (Fig. 7; partial Spearman's correlation analysis removing the influence of the subject). These unexpected results might be due to our stimuli. The rotation of the wedge and the rapid and continuous change of the mask pattern might have prevented the local adaptation process. We speculate that adaptation in early visual areas might not play a key role in the perceptual alternation for our stimuli. Other factors, such as neural noise in early visual areas, as suggested in previous studies (Brascamp et al. 2006), adaptation in higher brain regions or feedback from fronto-parietal network might play significant roles.

Eye movements and resulting retinal image change have been reported to contribute to perceptual switch in BR (van Dam and van Ee 2006). It is, however, unlikely that eye movements during the scans caused or severely affected the observed correlation for the following reasons. First, as all subjects were experienced psychophysical observers and had participated in the retinotopic mapping experiment, it was not hard for them to maintain fixation during the scan. Therefore, it is unlikely that they made large and frequent saccades that would evoke large responses, as previously reported (Kimmig et al. 2001). Second, eye movements during binocular rivalry predict correlations in the opposite direction as those we observed. Specifically, eye movements would shorten the suppression duration (van Dam and van Ee 2006) and, at the same time, they would shift the retinal location of the wedge and, consequently, weaken the retinotopic responses to the wedge. Therefore, if perceptual switches were mainly triggered by eye movements, a positive correlation between the fMRI responses and the suppression durations would have been observed; this is opposite to the observed result. Of course, we cannot fully rule out the possible unknown effects of eye movements since we did not measure eye movements during the scan. It is also possible that similar neural processes underlie eye movements under natural viewing and perceptual alternation during BR, as suggested by Hancock et al. (2012).

We used CFS as a tool to probe BR dynamics, treating CFS as a special case for BR, based on the fact that both CFS and BR involve interocular suppression and exhibit perceptual alternation. In fact, most of our subjects experienced many perceptual alternations instead of constant suppression during the prolonged exposure to the CFS stimulus as long as 6 min. However, psychophysical studies have shown that the dynamics and strength of CFS are qualitatively and quantitatively different from those of conventional BR, suggesting that “CFS is not a stronger version of BR” (Tsuchiya and Koch 2005; Tsuchiya et al. 2006). If these differences originated from the distinctiveness of the switching mechanisms between CFS and BR, then our observations using CFS might not be generalizable to conventional BR.

The observed negative correlation between the magnitude of retinotopic signals and suppression durations has two important implications for the neurophysiological underpinnings of visual awareness. The first implication is that, for something to be visible, a stable retinotopic representation is required. Consistent with this, the TMS study by Pearson et al. (2007) demonstrated the retinotopic specificity of TMS interference on BR. The significance of retinotopic representations for visual awareness has been evidently shown by an fMRI study of metacontrast masking (Maeda et al. 2010).

The second implication is related to one of the simplest assumptions in biological theories of consciousness, termed “activation thresholds.” According to this theory, any neural activity that satisfies a certain sufficient condition (e.g., the amount of activity, the duration of activity, or other factors) will produce consciousness of the content it represents (Palmer 1999). Although this assumption is too simple to be entirely true (see, Rees 2007), it is generally thought that conscious representations are stronger than unconscious ones (e.g., Cleeremans 2008). Given the idea of activation thresholds, a parsimonious interpretation of the negative correlation would be that the weaker the unconscious cortical representation is, the less likely it is to produce consciousness, because it is far from the threshold of visual awareness. Importantly, in the present study, the association was found across individuals. This may imply that the activation threshold for awareness would remain nearly constant across individuals. Otherwise, the association between the brain activity and the invisibility would be confined within an individual.

## GRANTS

This study was supported by a Grant-in-Aid for Scientific Research on Innovative Areas “Shitsukan” from the Ministry of Education, Culture, Sports, Science and Technology of Japan (23135517 and 25135720 to H. Yamamoto; 23135515 and 25135719 to J. Saiki), Grants-in-Aid for Scientific Research from the Japan Society for the Promotion of Science (JSPS; 22530793 to H. Yamamoto; 24240041 and 24650135 to J. Saiki), and Grant-in-Aid for Research Activity Start-up (22830039) from JSPS (to H. Yamashiro).

## DISCLOSURES

No conflicts of interest, financial or otherwise, are declared by the author(s).

## AUTHOR CONTRIBUTIONS

Author contributions: H. Yamashiro, H. Yamamoto, and J.S. conception and design of research; H. Yamashiro, H.M., M.U., and T.H. performed experiments; H. Yamashiro analyzed data; H. Yamashiro and H. Yamamoto interpreted results of experiments; H. Yamashiro prepared figures; H. Yamashiro and H. Yamamoto drafted manuscript; H. Yamashiro and H. Yamamoto edited and revised manuscript; H. Yamashiro, H. Yamamoto, H.M., M.U., T.H., and J.S. approved final version of manuscript.
